# Internal reference for determining liquid crystal orientation at alignment layers in liquid crystal cells by confocal polarised Raman microscopy

**DOI:** 10.1039/d5cp03926f

**Published:** 2026-01-26

**Authors:** Ruben Feringa, J. M. Bas Klement, Jasmine S. Sears, Pieter J. van der Zaag, Wesley R. Browne

**Affiliations:** a Stratingh Institute for Chemistry, University of Groningen Nijenborgh 3 9747 AG Groningen The Netherlands w.r.browne@rug.nl; b Reality Labs Research Meta, Redmond Washington USA; c Zernike Institute for Advanced Materials, University of Groningen Nijenborgh 3 9747 AG Groningen The Netherlands

## Abstract

The transmission of light through liquid crystal (LC) displays is controlled by reversible switching of the alignment of a mesogen using electric fields. In the absence of an electric field, the orientation of the mesogens is controlled by the layer of polymer, rubbed unidirectionally, on an ITO (indium titanium oxide) electrode on glass. The realignment induced by an applied electric field, to switch a pixel, is inefficient close to the solid liquid interface where the alignment layer has greatest interaction with the LC molecules and thereby reduces the darkness that can be achieved with LC display pixels. Characterising changes in orientation of liquid crystal molecules, *e.g.*, 5CB, at the alignment layer/LC interface is potentially possible by making use of the polarisation dependence and spatial resolution of confocal Raman microspectroscopy (CFRM). However, the optical properties, *e.g.*, refractive index, of the LC phases are dependent on LC orientation also, which limits control over spatial (depth) resolution in CFRM. Here, we introduce a resonance Raman active component, [Fe(bipy)_3_](BArF)_2_, into a PMMA alignment layer as an isotropic internal reference for CFRM. The Raman scattering from this compound is insensitive to the direction of polarisation of the excitation laser and enables estimation of the confocal depth probed in complete liquid crystal cells under operation. This layer enables changes in the depth of focus, due to change in refractive index, to be determined in real time when a potential is applied across the LC cell. This reference approach enables following the alignment of mesogens at the solid/LC interface in real time.

## Abstract 2

A polarisation insensitive label in the alignment layer of a liquid crystal (LC) cell enables sensing of the changes in optical transmission to be tracked as the alignment of a mesogen is switched with electric fields.

## Graphical abstract

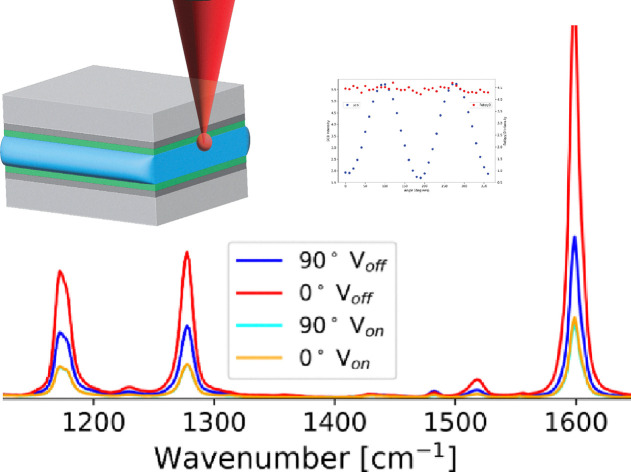

## Introduction

Liquid crystals (LCs) are used in applications ranging from displays and tuneable optical filters to adaptive materials.^1^ The response of liquid crystals to external stimuli, in particular to electric fields, temperature, light *etc.*, is the basis for these applications.^2^ The orientation/phase of a liquid crystal can be tuned precisely by, for example, adding (chiral) dopants,^3^ or contact with a so-called alignment layer.^4^

LCDs (liquid crystal displays) are based on the twisted nematic phase induced by alignment layers – thin layers of polymers that are scratched along one direction by rubbing. Indeed, as Ishihara *et al.*^4^ noted pertinently, “the history of LCD development is the history of alignment control of LC molecules”.

**Fig. 1:**
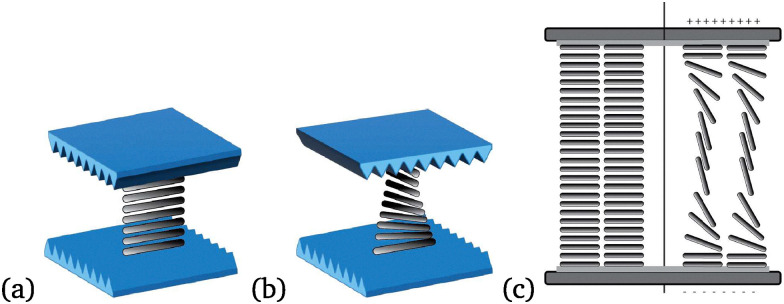
Liquid crystal alignment in an LC cell (a) in parallel and (b) perpendicular orientation, and (c) and persistence of the alignment at the alignment layer when an electric field is applied.

The operation of LCD pixels depends on control over the orientation of liquid crystal molecules and the effect these ordered orientations have on the transmission of linearly polarized light. The molecules at the interface of the rubbed polymer layer align in the direction of rubbing and this alignment propagates into the LC layer. A twisted nematic phase is formed when the top electrode has an alignment orthogonal to the bottom electrode (Fig. 1).

The liquid crystal molecules reorient when an electric field is applied, the Fréedericksz transition, resulting in switching of transmission of polarised light (off state) (Fig. 1). The reorientation is incomplete as the electric field has to overcome molecule/alignment layer interactions close to the interface. The extent of realignment governs to a large extent the contrast ratio of LCD screens.^5^ A complete off (dark) state is crucial in display/television applications and the lack of this in LCD displays makes them less competitive in terms of picture quality compared to emissive display technologies, such as OLED. As the interactions between the alignment layer and the liquid crystal determines the orientation of liquid crystal molecules, improving performance of the alignment layer would benefit from knowledge of the orientation near the alignment layer with good spatial resolution.

Non-destructive/-invasive imaging techniques sensitive to the orientation are necessary to study the behaviour of molecules at the LC/alignment layer interface.^6^ Several commonly available techniques enable determination of the orientation of liquid crystals, such as cross polarised microscopy and polarised transmittance spectroscopy.^7^ Indeed, polarised transmission is a gold standard in quantifying the performance of liquid crystal display devices, and the orientation of the molecules through which polarised light travels before passing through the second polariser. However, the observed transmittance is the net result of the contributions of the LC over the whole path length. Hence the technique does not provide information as to the spatial distributions of orientations, especially at the alignment layer.

Raman spectroscopy is particularly suited to determine the spatial orientation of the liquid crystal molecules.^8,9^ Raman scattering gives a fingerprint of the liquid crystal and the band intensities depend on the direction of polarisation of the laser. The Raman spectra of liquid crystalline compounds can be informative not only of structure but also orientational anisotropy (bulk ordering in an LC phase), due to the polarisation dependence of the Raman scattering. Unaxial cyano biphenyl LCs, for example 5CB and 8CB, show highly anisotropic Raman scattering (polarisability) tensors, which is manifested in variation in intensity depending on their orientation with respect to the direction of polarisation of the laser.^9^ The change in intensity of the nitrile stretching band (∼2227 cm^−1^) and carbon–carbon stretching band of aromatic rings (∼1607 cm^−1^) is large when the laser polarisation is parallel to the direction of the long axis of the LC molecules compared to when it is orthogonal. The in-plane deformation band of alkyl chain C–H bond (∼1180 cm^−1^) and the intensity of the Raman bands due to the stretching mode of benzene ring carbon–carbon bonds (∼1286 cm^−1^) are less sensitive to the molecular orientation.^10^ Raman spectroscopy is therefore also used to determine the configuration of the liquid crystal molecules^8^ or to observe temperature dependent phase transitions between oriented and isotropic states.^11^ For example, thermal phase transitions induced by a 1064 nm laser were monitored in real time by Raman microspectroscopy at 532 nm (1064 nm and 532 nm were combined into a single path at the sample) by Usman *et al.*^12^ The alignment of the liquid crystal molecules is disrupted when heated sufficiently by the 1064 nm laser to form an isotropic phase as a “ghost particle”, which is manifested in a change in Raman scattering intensity of specific bands.^12^

Raman spectroscopy is inherently confocal and hence Raman scattering from the liquid crystal in an LCD depends on the position of the focal point of the objective, which coupled with polarised light, can provide information on orientation of the liquid crystal with respect to the optical axis of the Raman microscope, *i.e.* confocal polarized Raman microscopy (CPRM). CPRM has been used to probe liquid crystal orientation to detect the presence of nickel ions at the solid LC interface.^13^

The interface formed between the liquid crystal and the alignment layer determines the behaviour of the liquid crystal for some distance from the surface, and hence understanding the interaction and the orientation of LCs at this interface is important in improving alignment layer function in LC applications. Ekgasit *et al.* applied the surface sensitive polarised-FTIR-ATR spectroscopy to study changes at the alignment layer in LC cells.^14^ Buyuktanir *et al.* reported the electric field dependent orientation of liquid crystals in an LC cell using polarized Raman spectroscopy, using interdigitated ITO electrodes.^6^ The electrodes induce orientation along a curved electric field to create a bow shaped liquid crystal phase. The arrangement of the electrodes, however, result in reorientation of the LC in a plane orthogonal to the transparent path of the cell. Ideally, the changes induced in a standard LCD cell format, where the reorientation is in a plain parallel to the optical axis, would be followed. However, the birefringence of the liquid crystal material means that it is challenging to be certain of the confocal depth of the Raman microscope used due to induced defocusing. The birefringence results in distorted images of the selected volume and therefore is not a reliable technique. Raman active alignment layers offer a tool to account for defocusing (loss of depth resolution) and changes in focal position in LCD cells.

**Fig. 2:**
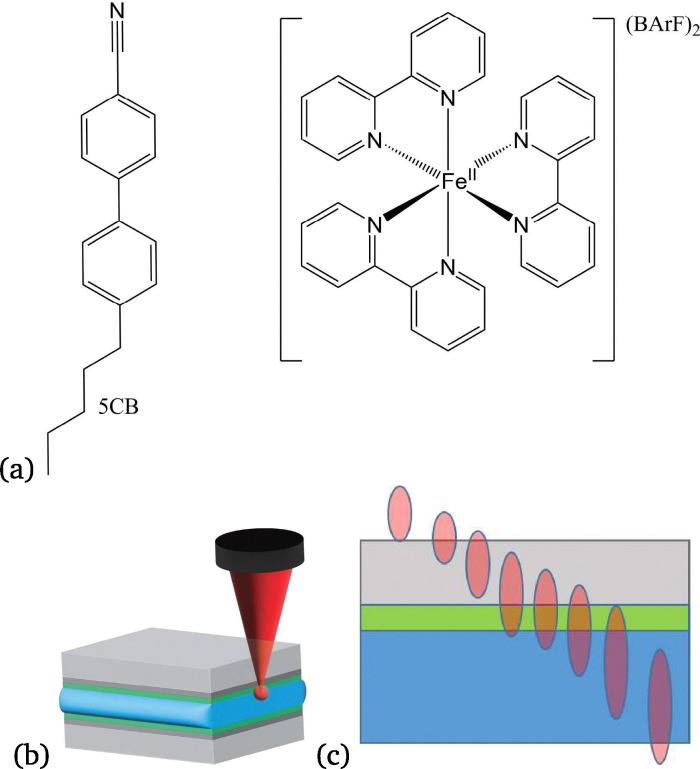
(a) Structures of 5CB and [Fe(bipy)_3_](BArF)_2_ and (b) ITO (dark gray) on glass (light gray) liquid crystal (blue) cell with Raman active alignment layer (green). (c) Movement (and axial elongation) of confocal volume of microscope from the glass over the alignment layer to the liquid crystal. [Fe(bipy)_3_](BArF)_2_ is dispersed in the PMMA alignment layer indicated as green in the images.

Here, we show that the resonance Raman active compound [Fe(bipy)_3_](BArF)_2_ (where bipy is 2,2′-bipyridyl and BArF^−^ is tetrakis(3,5-(trifluoromethyl)phenyl)borate) added to PMMA alignment layers provides an isotropic Raman spectrum that can be used as a spatial reference. The Raman scattering from this compound is insensitive to laser polarisation and enables detection of changes in the confocal depth of the Raman microspectrometer during the operation of LC cells. We use the Raman bands of this compound to track changes in confocal depth during electric field induced switching of an LC cell. With this approach the orientation of molecules at the alignment layer and in the bulk can be studied (Fig. 2).

## Results and discussion

### Raman spectroscopy of PMMA/[Fe(bipy)_3_]^2+^ alignment layers

PMMA is a less common alignment layer for LC cells. However, its spectrum shows limited overlap with those of the 5CB and of [Fe(bipy)_3_](BArF)_2_. Resonance enhancement of the Raman scattering of [Fe(bipy)_3_](BArF)_2_ means that it can be observed at sub-millimolar concentrations at *λ*_exc_ 532 nm (Fig. S3).^15^ Moreover, it does not show phosphorescence that would otherwise interfere with the collection of Raman spectra.^16^ Furthermore, the pretilt angle and anchoring of PMMA is different from, *e.g.*, polyimide, polymer alignment layers, lacking aromatic interactions with 5CB and hence alignment is driven purely by polar/apolar interactions.

Spin coating of PMMA, and subsequent rubbing to create the alignment layer, can result in polarisation dependent Raman scattering from the alignment layer itself. A key advantage of using the resonant Raman active complex [Fe(bipy)_3_](BArF)_2_,^17^ as a dopant in the PMMA coating, therefore, is that it provides Raman scattering that is insensitive to the polarisation of the laser.^18^ [Fe(bipy)_3_](BArF)_2_ is a pseudo-octahedral complex due to the bidentate 2,2′-bipyridyl ligands and has low symmetry. Hence, when in solution or immobilised in PMMA, its Raman spectrum is insensitive to the direction of polarisation of the excitation laser, as demonstrated by comparison of the Raman spectra obtained in PMMA films and in ethyl acetate (Fig. S3).

Raman spectra of doped PMMA layers (Fig. S3) show bands of [Fe(bipy)_3_](BArF)_2_ that are identical to those of the complex both in powder form and in ethyl acetate solution. The doping of [Fe(bipy)_3_](BArF)_2_ (*ca.* 0.8 mM) in the final layer was estimated by comparison of the relative intensity of the Raman scattering of the complex with that of the carbonyl stretching band of the PMMA/ethylacetate (Fig. S2).

The doped alignment layer showed the same physical properties, *i.e.* negligible visible absorption, and the same water contact angle, as PMMA alone, which together with lack of leaching of the complex in brine (Fig. S2), indicates that the presence of the complex has a negligible effect on the properties of the PMMA coating as an alignment layer. The uniformity of the dispersion of the complex in the PMMA film was determined by Raman spectroscopy (Fig. S4).

**Fig. 3:**
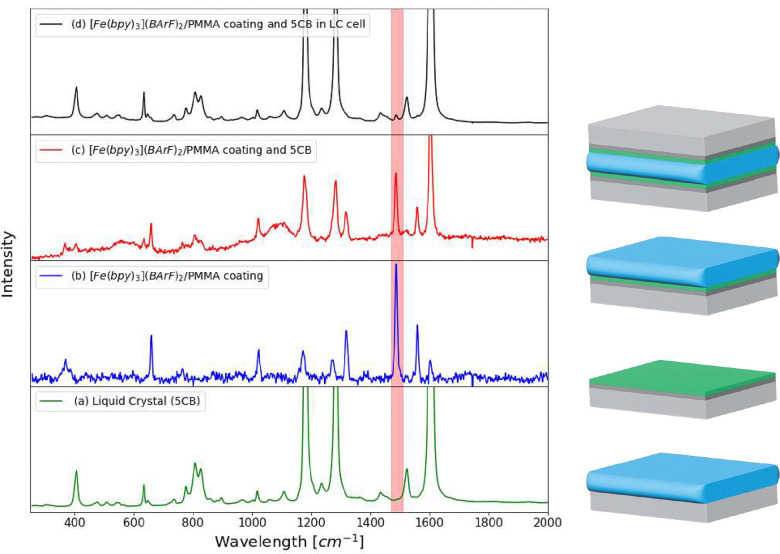
Left: Raman spectra of (a) neat 5CB, (b) PMMA film with [Fe(bipy)_3_](BArF)_2_, (c) PMMA film with [Fe(bipy)_3_](BArF)_2_ with a thin film of liquid crystal on top and (d) in a complete LC cell. A characteristic [Fe(bipy)_3_](BArF)_2_ band is highlighted in red. Glass (grey), ITO (dark grey), 5CB (blue), PMMA with [Fe(bipy)_3_](BArF)_2_ (green). Right: Scheme showing each sample: glass (light gray), 5CB (blue), ITO (dark gray), PMMA/[Fe(bipy)_3_](BArF)_2_ (green).

**Fig. 4:**
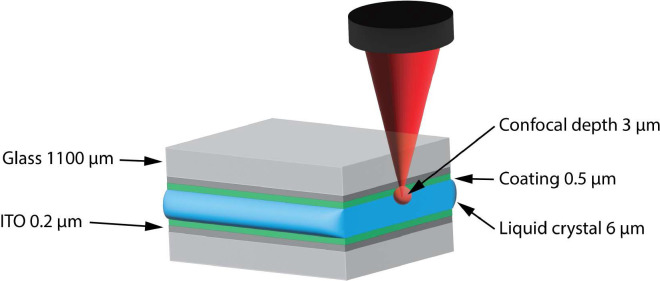
Liquid crystal cell with the dimensions of each of the layers. The 6 µm layer of LC is typical for LCDs.

The thickness of the coating used as an alignment layer in LC cells for further studies was chosen such that the [Fe(bipy)_3_](BArF)_2_ was readily observed by Raman spectroscopy, yet sufficiently thin to under-fill the minimum confocal depth (<3 µm) considerably. The thin layer ensures that Raman scattering from the alignment layer and from 5CB can be observed simultaneously. It should be noted that the thickness of the alignment layer also influences the minimum voltage that needs to be applied to induce the Fréederickz transitions.^19^ The Raman spectrum of the liquid crystal 5CB as a thin film (isotropic) on an ITO coated glass slide and on a slide coated with PMMA/[Fe(bipy)_3_](BArF)_2_ show that several bands from the complex do not overlap with bands of 5CB (Fig. 3). The band at 1486 cm^−1^ in particular is still observed (Fig. 3 and 6) in the fully assembled LC cell (Fig. 4). Hence, the [Fe(bipy)_3_](BArF)_2_ in the PMMA alignment layer is suitable as an isotropic internal reference. The depth selectivtiy that can be achieved in complete LC cells is addressed in the next section.

### Depth profiling of PMMA coated slides with [Fe(bipy)_3_]^2+^

**Fig. 5:**
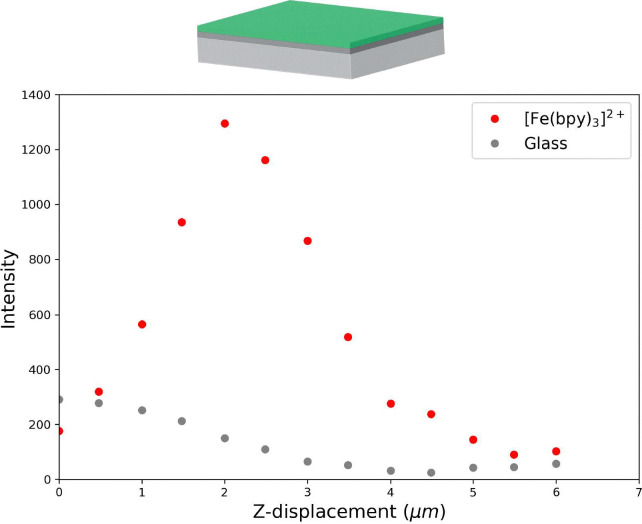
Intensity of Raman scattering from an ITO on glass slide coated with PMMA/[Fe(bipy)_3_](BArF)_2_ approached from PMMA coated side. The intensity of Raman scattering from glass (grey) and [Fe(bipy)_3_](BArF)_2_ (red) is shown as the sample is moved through the focal point of the objective. Positive z-displacement indicates depth through coating and glass slide.

The impact of the various components of the LC cell on the depth resolution of the microscope was determined by first depth mapping through a coating of PMMA/[Fe(bipy)_3_](BArF)_2_ on top of an ITO on glass slide. A 25 µm pinhole was used to maximise depth resolution (Fig. S1). The intensity of the Raman bands of glass compared with the intensity of the resonantly enhanced Raman band of [Fe(bipy)_3_](BArF)_2_ at 1486 cm^−1^ (Fig. 5) show a dependence on depth of the confocal volume in the cell as expected. The Raman scattering from the glass substrate decreases as the focal point of the objective passes through the glass and then into the PMMA coating. The intensity of the bands of [Fe(bipy)_3_](BArF)_2_ increase and then decrease over roughly 3 µm, which is within that expected from the calculated confocality of the system (*ca.* 3 µm) and the thickness of the doped PMMA film (0.58 µm).

**Fig. 6:**
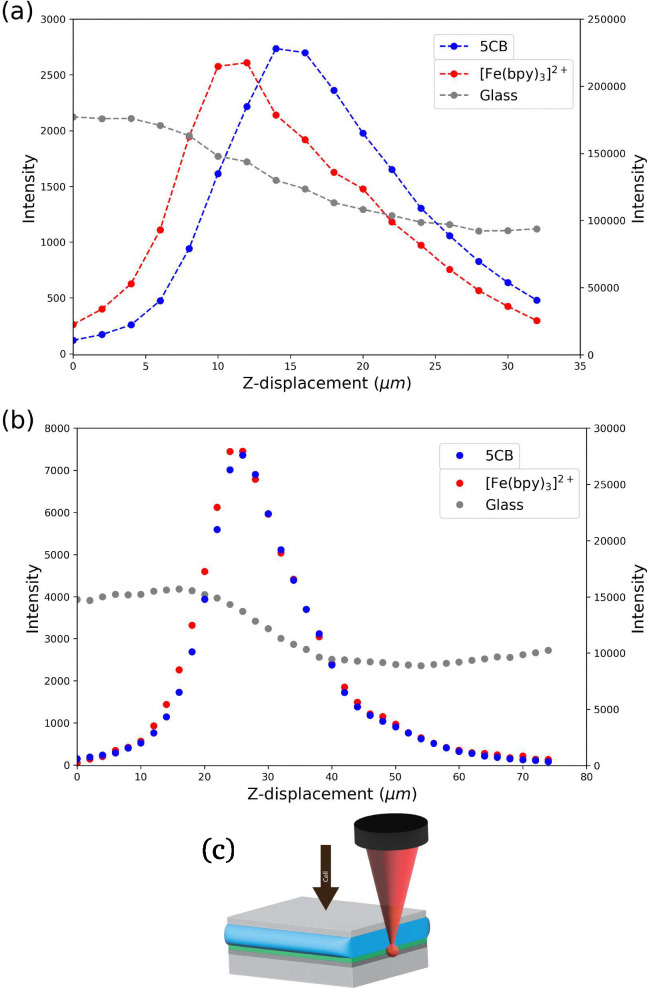
Z-mapping of a liquid crystal cell with (a) a 25 µm, and (b) a 100 µm pinhole. (c) The cell is comprised of a layer of glass coated with PMMA/[Fe(bipy)_3_](BArF)_2_ layer, with a 6 µm spacer and a cover slip on top. Intensity of Raman scattering of glass (gray), [Fe(bipy)_3_]^2+^ (red) and 5CB Raman bands (blue) over a range of depths, measured through the top of the cell. Positive z-displacement indicates depth into the LC cell.

In contrast, when measuring the PMMA coating through the ITO on glass, the effects of the difference in refractive index between glass and air on depth confocality become apparent (Fig. 2).^20^ The Raman scattering of [Fe(bipy)_3_](BArF)_2_ was *ca.* 80% weaker, and the depth confocality was reduced to *ca.* 20 µm, *i.e.* greater that the thickness of the LC cell (Fig. S9).^21^ Using ITO coated cover slips (0.15 mm thick), which is much thinner, helps to reduce this aberration making it possible to distinguish the Raman bands of [Fe(bipy)_3_](BArF)_2_ from the Raman bands of the liquid crystal (*vide infra*, Fig. 6).^22^

### Polarisation dependence of Raman scattering of 5CB in linear and twisted LC cells

The intensity of Raman scattering of 5CB depends on the orientation of the molecules of 5CB with respect to the polarisation of the incident laser. As expected from its low symmetry, the depolarisation ratios for all Raman bands of 5CB are the same (Fig. S6). Hence, for an isotropic solution of 5CB, the direction of polarisation of the laser does not make a significant difference to the absolute and relative intensities of the Raman bands. With a twisted nematic LC cell the Raman scattering is essentially constant regardless of the direction of polarisation of the laser propagating through the LC cell (Fig. S8).

### Raman microscopy with [Fe(bipy)_3_](BArF)_2_ and 5CB

The Raman spectrum of 5CB shows several intense bands characteristic of the functional groups present, in particular the nitrile and biphenyl stretching bands, as well as weaker bands related to the aliphatic chain (Fig. 3). The Raman bands of [Fe(bipy)_3_](BArF)_2_ that show resonantly enhanced Raman scattering are those associated with the 2,2′-bipyridyl ligand. Positioning the interface of the liquid crystal and PMMA alignment layer within the focal volume of the objective, enables the observation of Raman bands of both [Fe(bipy)_3_](BArF)_2_ and 5CB simultaneously (Fig. 3) using a glass cover slip as the top half of the cell (Fig. 6). With a 100 µm pinhole in the optical path to the spectrometer the depth spatial resolution was insufficient to distinguish the two layers, and indeed bands of [Fe(bipy)_3_]^2+^ were observed over the same range of depths as the 6 µm layer of 5CB despite that the PMMA layer was *ca.* 0.5 µm^23^ thick. With a 25 µm pinhole (Fig. 6), the intensity of Raman scattering from [Fe(bipy)_3_](BArF)_2_ increases and decreases as the interface passes through the focal plane of the microscope as do the bands of 5CB. However, the depth over which the intensity of each bands changes is different. The point at which the intensity of the Raman bands of [Fe(bipy)_3_]^2+^ are maximum was selected as the position of the interface of the alignment layer and the liquid crystal.

### Orientation of the liquid crystal in a linear LC cell

**Fig. 7:**
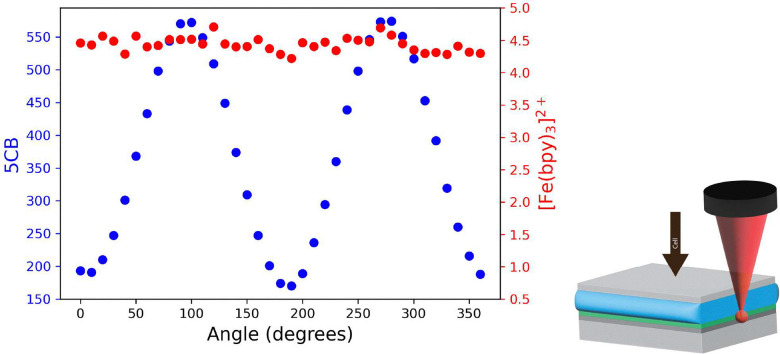
Intensity of the bands of [Fe(bipy)_3_](BArF)_2_ (red) in a PMMA (0.5 µm layer), on the bottom electrode of an LC cell (6 µm thick), and of the 5CB (blue) under rotation of the polarisation of the excitation laser (532 nm, with 100 µm collection pinhole). An ITO coated coverslip served as the top electrode (objective side).

In a linear LC cell, *i.e.* where both PMMA covered electrodes are rubbed in the same direction, the equilibrated state of the LC molecules is to align with the direction of the alignment layer through the whole cell. We use this cell to contrast the polarisation dependence of the Raman scattering of 5CB and the insensitivity to laser polarisation direction of the Raman scattering of [Fe(bipy)_3_](BArF)_2_. A constant intensity of Raman scattering of [Fe(bipy)_3_](BArF)_2_ was observed, while the intensity of the 5 CB bands oscillated according to the angle of polarisation of the laser, as expected for linear alignment at the PMMA/5CB interface (Fig. 7). It is of note that in contrast to most of the bands the intensity of the Raman band of 5CB at 625 cm^−1^ does not show sensitivity to the direction of polarisation of the laser and hence can be used for spectral intensity normalisation (Fig. S8, *vide infra*).

### Application

#### Raman microspectroscopy in LC cells

The polarisation dependence of the Raman scattering intensity on the orientation of liquid crystalline molecules can be used to report changes in orientation upon application of an electric field over the liquid crystal cell with alignment layers, *i.e.* the Fréederickz transition. As discussed above, the applied electric field must overcome the alignment induced by the alignment layer. The potential gradient needed to induce full reorientation decreases away from the electrode surface as a result. However, the difference in refractive indices^24^ of the LC and air *etc.* reduces the depth resolution and hence the precise volume and depth probed during depth mapping.^25^

A LC cell with a 0.5 µm PMMA/[Fe(ii)(bipy)_3_](BArF)_2_ alignment layer on the bottom (with respect to the microscope objective) of the LC cell, a bare ITO-coated quartz coverslip as top electrode and a 6 µm layer of 5CB (*i.e.* similar to that studied in Fig. 6), was used to study the effect of electric field at different depths in the cell. The bottom electrode, with the alignment layer, was focused on using the Raman bands of [Fe(ii)(bipy)_3_](BArF)_2_, to determine the orientation of laser polarisation at which the maximum in Raman scattering from 5CB is observed near the lower surface (Fig. S8).

The intensities of Raman scattering from 5CB and from [Fe(ii)(bipy)_3_](BArF)_2_ vary differently with depth of the confocal volume with respect to the LC cell, Fig. S10. The cell has an alignment layer on only one electrode and hence the ordering of 5CB is expected to decrease away from that electrode. Indeed the Raman intensity (normalised at 625 cm^−1^) of the polarisation sensitive bands of 5CB (at 1600 cm^−1^) is lower when the focus is closer to the top non-coated electrode compared with the intensity near the PMMA/[Fe(ii)(bipy)_3_](BArF)_2_ coated electrode, Fig. 9. These differences are consistent with the expected difference in ordering of the 5CB at the top and bottom layers.

#### Potential driven LC phase transitions

**Fig. 8:**
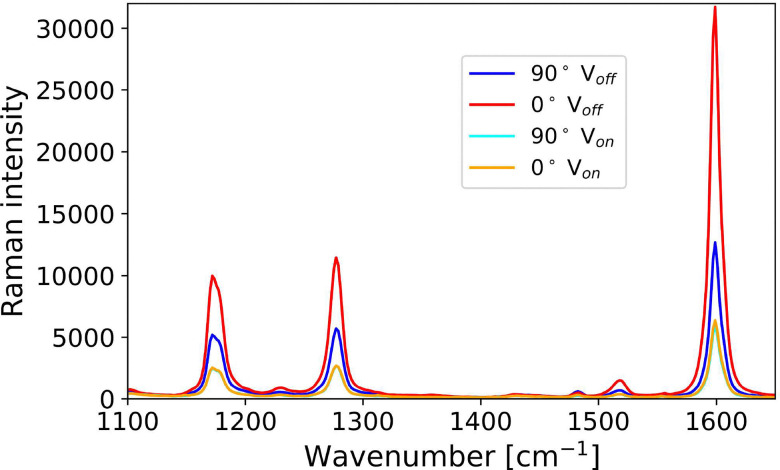
Confocal Raman spectra of a liquid crystal cell using a 25 µm pinhole with and without application of an alternating voltage across the cell. The cell configuration is as in Fig. 6. Spectra recorded with polarisation of laser along (0°, red and orange) and orthogonal (90°, blue and cyan) to the axis of alignment. Spectra are normalised to the band at 625 cm^−1^.

The effect of alternating voltage (60 Hz, 10 V) on the Raman spectrum of the LC cell was determined at a depth of 0 µm, defined as the point of the maximum intensity of the Raman bands of [Fe(ii)(bipy)_3_](BArF)_2_, and secondly with the confocal volume 13 µm^26^ above this layer (where only bands of 5CB were observed). The polarisation of the laser was rotated so that a maximum in Raman intensity of the 5CB was obtained at 0 µm and then at 90° to that orientation, without and with an applied voltage (Fig. 8). A large change in the Raman intensity of the 5CB bands is observed between 0 and 90°. Application of an alternating voltage resulted in an overall decrease in intensity (due to change in refractive index, *vide infra*) and a loss in the dependence of Raman intensity on the direction of polarisation of the laser.

**Fig. 9:**
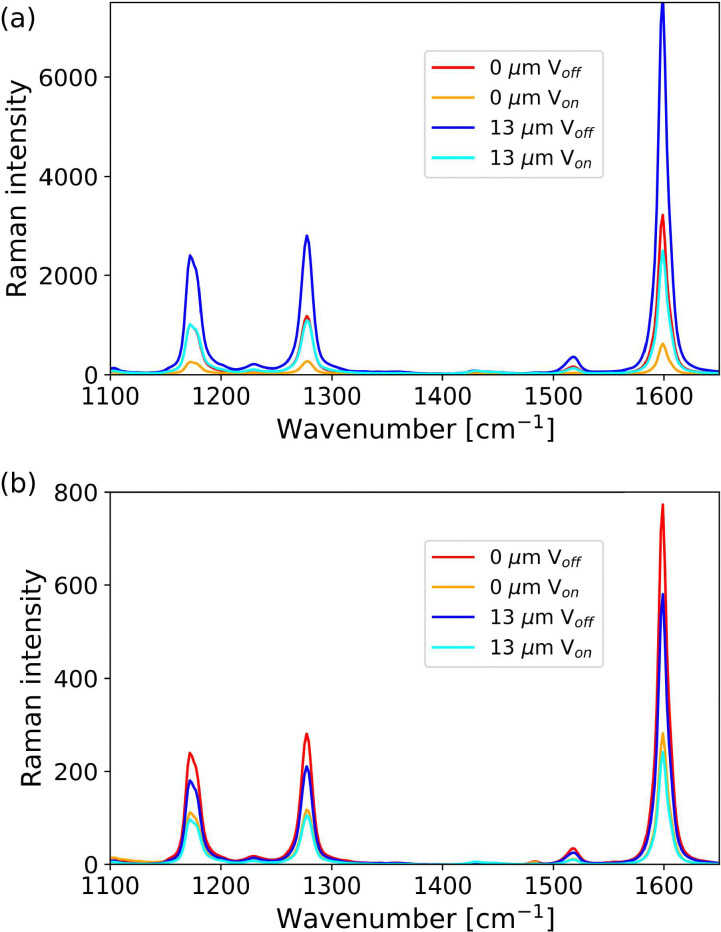
Confocal Raman spectra of a liquid crystal cell using a 25 µm pinhole. The cell configuration is as in Fig. 6. Spectra recorded with polarisation of laser along (0°) the axis of alignment at 0 and 13 µm depths. Spectra before (a) and after (b) normalisation to the band at 625 cm^−1^. See Fig. S15 for full spectra.

The switching ratio (electric field off/on) obtained from the absolute intensity of the 5CB Raman bands at 0 and 13 µm is 5.13, and 3.05 (Fig. 9). The lower ratio at 13 µm would be consistent with less initial alignment near the top electrode already before the electric field is applied. However, normalisation of the spectra, at the polarisation insensitive band of 5CB at 625 cm^−1^, shows that the differences observed are mostly due to the change in optical properties (refractive indices) and the changes in intensity are similar at both depths (Fig. 9).

Notably, the intensity of Raman scattering from [Fe(ii)(bipy)_3_](BArF)_2_ changes also when an electric field is applied. The change in intensity reflects the change in depth of focus expected due to the change in the refractive index of the 5CB^27^ as its bulk orientation is changed. The decrease in the [Fe(ii)(bipy)_3_](BArF)_2_ bands emphasises an important benefit of the approach taken here, as it enables the change in the z-position of the confocal volume upon switching of the LC to be detected in real time. A further point to note is that the wavelength dependence of the refractive index of 5CB is relatively steep^28^ and hence it is an advantage that the reference bands from [Fe(ii)(bipy)_3_](BArF)_2_ are close to the 5CB band of interest.

## Conclusion

Using alignment layers with resonance Raman active components enables the study of interactions and molecular orientation at the solid–liquid crystal interface. [Fe(bipy)_3_](BArF)_2_ is such a Raman active component that can be dissolved in ethyl acetate/PMMA and spin coated on an ITO coated glass slide. Its Raman scattering is isotropic (independent of laser polarisation), which facilitates the study of the interface of the alignment layer and the 5CB liquid crystal. Raman scattering from liquid crystals depends on the polarisation of the incident light, due to the anisotropy of the liquid crystal caused by the alignment layer. Although the orientation of LC molecules can be discerned using polarized Raman microscopy, the isotropic Raman scattering of [Fe(bipy)_3_](BArF)_2_ enables the effect of changes in the optical path of the laser to be identified, such as the effects of glass and refractive index changes. Applications of this approach that can be envisaged are, for example, where dopants are added to the liquid crystal, to distinguish the effect of dopants at the interface of the alignment layer and in the bulk LC phase. This approach can potentially be used to study temporal as well as spatial effects, such as response times to voltage switching and switching frequency. For these applications, the time resolution achievable is limited only by signal to noise ratio achievable with the optical system and the spectrometer/detector used.

## Experimental

### Materials, compounds, chemicals and consumables

5CB liquid crystal was used without further purification (synthon chemicals GmbH&Co). PMMA was used without further purification (mw ∼120 000, Sigma-Aldrich). ITO coated glass slides (CEC007S) were purchased from Prazisions Glas & Optik GmbH, Germany, and ITO cover slips (UQG Part No. CIO-1858) from UQG Optics UK.

### Synthesis of [Fe(bipy)_3_](BArF)_2_

[Fe(bipy)_3_](BArF)_2_ was prepared by mixing aqueous solutions of iron(ii) sulfate and 2,2′-bipyridine in a 1 : 3 molar ratio yielding an intense red solution, followed by addition of 2 equivalents of sodium tetrakis(3,5-(trifluoromethyl)phenyl)borate (NaBArF) in methanol. The red precipitate was collected by filtration, washed with water and then methanol.^29^

### Instrumentation

UV/vis absorption spectra were recorded using a Specord600 UV/vis spectrophotometer. Raman spectra at 785 nm were recorded using a RamanStation 400F equipped with polarisation optics (PerkinElmer) or a Raman microscope as described elsewhere.^30^ AC switching of the LC cells was carried out using a Tetronix signal generator. The choice of excitation wavelength, 532 nm, for the present study was motivated by several practical considerations. Other common, wavelengths such as 632.8 nm and 785 nm were considered, however, spontaneous emission from the 5CB at 632.8 nm excitation and interference from emission from traces of Nd^3+^ in the glass substrate at 785 nm excitation (SI), together with the greater sensitivity of the detector in the visible region, prompted us to focus in the current study on excitation at 532 nm. Furthermore, 532 nm is an optimum wavelength for resonance enhancement of the Raman scattering of [Fe(ii)(bipy)_3_]^2+^, since the absorbance and hence potential secondary inner filter effects, decreases rapidly over the wavelength range of interest (*ca.* 540 to 640 nm, Fig. S5) for Raman scattering. The construction of the Raman microspectrometer is described in the experimental section. The beam expander and 
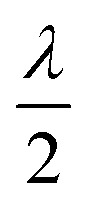
 retarder improves depth resolution (z-confocality) and control over the direction of polarisation of the excitation laser in the *x*/*y* plane, respectively. A broad band polariser was used before focusing of light into the spectrometer to characterise the polarisation dependence of the optical system, using isotropic droplets of 5CB (Fig. S6) and cyclohexane (Fig. S7) with reference to a corrected Raman spectrometer (see SI for details).

### Spincoating of PMMA

PMMA films containing [Fe(bipy)_3_](BArF)_2_ were prepared by spin coating ITO coated glass slides with solutions of the [Fe(bipy)_3_](BArF)_2_ (0.326 mM) at various concentrations of PMMA (*M*_w_ 120 kDa) in ethyl acetate using a Laurell WS-650MZ-23NPPB spincoater in static mode with, (1) 2 s at 400 rpm (acceleration 400 rpm), (2) 1.5 s at 1500 rpm (acceleration 1500 rpm), and (3) 30 s at 4000 rpm (acceleration 1200 rpm) unless stated otherwise.^31^ The PMMA layers were made by spincoating from ethyl acetate. Ethyl acetate does not provide for the smoothest layers of spincoated PMMA but is used because it will dissolve the [Fe(bipy)_3_](BArF)_2_ complex. The dimensions of the cell is important to note. The concentration of [Fe(bipy)_3_](BArF)_2_ in the film is too low to be determined by UV/vis absorption spectroscopy. However, an estimate can be made by comparing the carbonyl stretching band of PMMA which have a similar cross-section to ethyl acetate with the Raman bands of the complex. Comparison of the spectrum of a film prepared with 6 wt% with a solution of the complex in ethyl acetate (Fig. S2 and S3) suggestions the concentration is *ca.* 0.5 to 1 mM in the film or *ca.* 0.5 wt% in the film. The glass slide is 1100 µm with an 0.2 µm ITO layer on this layer a 0.5 µm layer of PMMA containing [Fe(bipy)_3_](BArF)_2_ is spincoated and a 6 µm spacer results in a 6 µm layer of 5CB (Fig. 4).

The thickness of the PMMA layers was determined by profilometry on a (Bruker DektakXT),^31^ from which a calibration curve was prepared (Fig. S11) for determination by FTIR absorption spectroscopy. FTIR spectra were recorded using a JASCO FTIR4700 spectrophotometer.

The stability of [Fe(bipy)_3_](BArF)_2_ in the PMMA films towards leaching was tested by soaking half of the PMMA layer on a glass slide in a solution of brine for one hour whereafter it was rinsed with demi-water and dried by contact with paper. Raman spectra before and after showed no difference, indicating that leaching was not significant (Fig. S2).

### Fabrication of LC cells

The liquid crystal cells were prepared using two ITO coated glass slides (CEC007S, Prazisions Glas & Optik GmbH, Germany). The ITO coated sides were coated with a layer of PMMA (*vide supra*) unless stated otherwise. The alignment layer was prepared by rubbing in one direction with a piece of cloth. Mylar spacer material with a thickness of 6 µm separated the pairs of ITO on glass slides, with either parallel or orthogonal arrangement with respect to the direction of alignment. The cell was fixed and sealed with photocurable glue (Thorlabs UV-curing Optical Adhesive Kit) before insertion of 5CB by capillary action.

## Author contributions

The manuscript was written through contributions of all authors. All authors have given approval to the final version of the manuscript.

## Conflicts of interest

There are no conflicts to declare.

## Supplementary Material

CP-028-D5CP03926F-s001

## Data Availability

The data supporting this article have been included as part of the supplementary information. The primary data is available on request from the authors. Supplementary information (SI): additional spectral data and description of the microscope. See DOI: https://doi.org/10.1039/d5cp03926f.

## References

[cit1] Lagerwall J. P., Scalia G. (2012). Cur. Appl. Phys..

[cit2] Andrienko D. (2018). J. Mol. Liq..

[cit3] Bala I., Plank J. T., Balamut B., Henry D., Lippert A. R., Aprahamian I. (2024). Nat. Chem..

[cit4] Ishihara S., Mizusaki M. (2020). J. Soc. Inf. Disp..

[cit5] Raman N., Hekstra G. (2005). IEEE Trans. Consum. Electr..

[cit6] Büyüktanir E. A., Zhang K., Gericke A., West J. L. (2008). Mol. Cryst. Liq. Cryst..

[cit7] Miller D. S., Carlton R. J., Mushenheim P. C., Abbott N. L. (2013). Langmuir.

[cit8] Castriota M., Fasanella A., Cazzanelli E., De Sio L., Caputo R., Umeton C. (2011). Opt. Express.

[cit9] Zhang Z., Panov V. P., Nagaraj M., Mandle R. J., Goodby J. W., Luckhurst G. R., Jones J. C., Gleeson H. F. (2015). J. Mater. Chem. C.

[cit10] Xin H., Chen H., Song P., Sun Q. (2023). Mater. Today Commun..

[cit11] Basumatary J., Gangopadhyay D., Nath A., Devi Thingujam K. (2023). Spectrochim. Acta, Part A.

[cit12] Usman A., Uwada T., Masuhara H. (2011). J. Phys. Chem. C.

[cit13] Rashid M., Singh S. K. (2023). Chem. Phys. Lett..

[cit14] Ekgasit S., Fulleborn M., Siesler H. W. (1999). Vib. Spectrosc..

[cit15] A point to note in regard to excitations at other wavelengths, *e.g.*, 632.8 nm, is that it is no longer resonant with the absorption spectrum of the complex. Other ligands that shift the 1MLCT absorption band to the red should then be used (*e.g.*, 2,2′-biquinoline)

[cit16] Juris A., Balzani V., Barigelletti F., Campagna S., Belser P., von Zelewsky A. (1988). Coord. Chem. Rev..

[cit17] [Fe(bipy)_3_](BArF)_2_ absorbs at 532 nm (Fig. S5)

[cit18] The BarF^−^ counterion was chosen to increase solubility in ethylacetate/PMMA mixtures used for spin coating

[cit19] Nemoto F., Nishiyama I., Takanishi Y., Yamamoto J. (2012). Soft Matter.

[cit20] Chakraborty S., Kahan T. F. (2019). J. Raman Spectrosc..

[cit21] A20 s exposure/5 accumulations was required to gain the same intensity compared with 5 s exposure/5 accumulations

[cit22] The 0.15 mm thick ITO coated glass coverslips are not strong enough to carry out spin coating on and hence were only used in the present study as uncoated second electrodes

[cit23] Attempts to increase the depth spatial resolution using immersion oil and a cover-slip corrected objective were unsuccessful due to the Raman scattering of the immersion oil at 532 nm

[cit24] Everall N. J. (2009). Appl. Spectrosc..

[cit25] Refractive index matching with an oil immersion objective does not eliminate this effect as the refractive index of the LC changes as orientation changes as discussed in the text

[cit26] The actual difference in depth is greater due to the effect of differences in refractive index air/glass/LC on the confocal depth probed by the microscope

[cit27] Datta Sarkar S., Choudhury B. (2010). Acta Phys. Pol. A.

[cit28] Horn R. (1978). J. Phys..

[cit29] Miller J. N., McCusker J. K. (2020). Chem. Sci..

[cit30] Klement W. J. N., Steen J. D., Browne W. R. (2023). Langmuir.

[cit31] Feringa R., Siebe H. S., Klement W. J. N., Steen J. D., Browne W. R. (2022). Mater. Adv..

