# Supplementary material for: Internal reference for determining liquid crystal orientation at alignment layers in liquid crystal cells by confocal polarised Raman microscopy
Source: Phys Chem Chem Phys. 2026 Jan 26;28(8):5337–44. doi: 10.1039/d5cp03926f (PMC12884478; doi:10.1039/d5cp03926f)
Supplement: CP-028-D5CP03926F-s001 [file CP-028-D5CP03926F-s001.pdf]

Supplementary Information (SI) for Physical Chemistry Chemical Physics.
This journal is © the Owner Societies 2026

Internal reference for determining liquid crystal orientation at alignment layers in liquid crystal cells by confocal polarised Raman microscopy

Ruben Feringa, J.M. Bas Klement, Jasmine S. Sears, Pieter J. van der Zaag, and Wesley R. Browne*

Electronic Supplementary Information

Instrumentation

Confocal Raman microscope

The collimated emission of a 532 nm (Cobolt Samba 25 mW) laser was directed through a mechanical shutter and thereafter the Keplerian beam expander. A silver mirror (Thorlabs PF10-03-P01) reflects the beam into a variable beam splitter (Thorlabs VA5-532/M) to control beam power and a $\frac{\lambda}{2}$ waveplate (Thorlabs WPHSM05-532) in a rotation mount (Thorlabs CLR1/M) directs the beam to a long pass dichroic mirror (Thorlabs B5CT1 + B4C/M Semrock LPD02-532RU-25) where after the beam is directed onto the optical axis of the spectrometer and into the microscope (Olympus BX-51), with a 100x objective (LMPlanFL N 100x/0.8) via two mirrors (Thorlabs BB1-E02). The collected Raman scattering travels through the long pass dichroic mirror and is focused through the pinhole (LA1805-ML plano-convex lens (f=30 mm) in a Thorlabs SM21ZA Z-axis translation mount with 2 mm travel, Thorlabs p100k 100μm pinhole). Thereafter the collimated beam is focused into the spectrograph (Andor Technology, Shamrock300i equipped with a 1200 l/mm grating blazed at 500 nm) and idus-420-BV CCD camera (Andor Technology). The sample was brought to focus by a manual sample stage equipped with on a piezoelectric controlled sample stage (Thorlabs LPS710E/M).

The laser focal spot-size was determined using an Edmund Optics Ronchi Ruler (240 l/mm) and a fluorescent slide. An image of the ruler was recorded on the microscope in brightfield mode using the 100x objective also used to record Raman spectra. The focal spot of the laser used in the Raman setup was measured by counting how many lines wide it is compared to a 240 l/mm ruling (figure S12). The focal spot is within 1 line, thus the diameter of the spot is $< 1/240$ mm = 4 μm, giving an area of <12 $(\mu m)^2$. The coating is 680 nm (IR absorbance of 0.046), which results in the scattering from a volume of 8.16 $(\mu m)^3$.

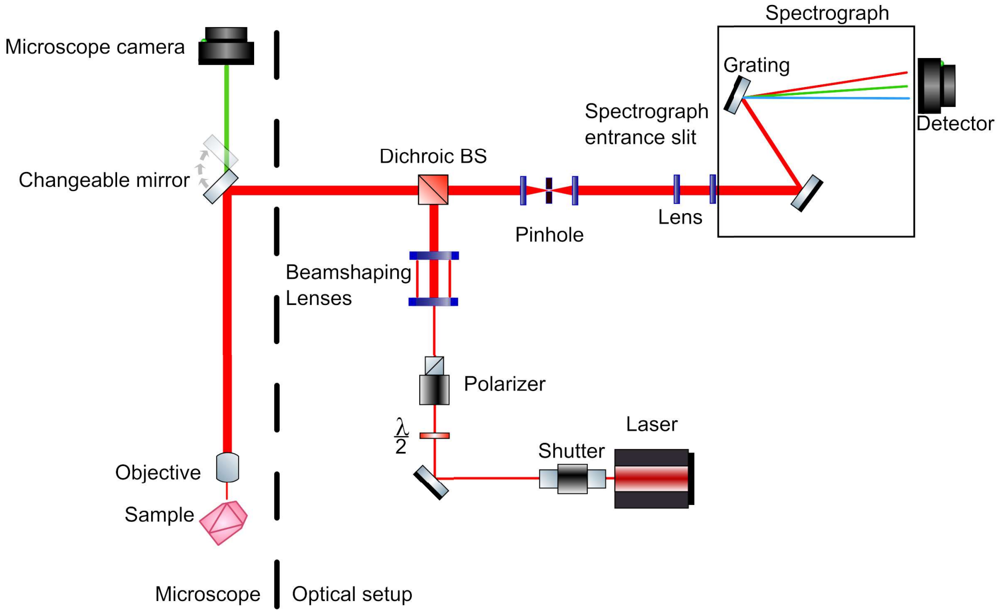

Fig. S1 Optical layout of polarized Raman microscope.

Depolarisation ratios and polarisation dependence of spectrometer

Depolarisation ratios for 5CB and cyclohexane were determined using a Perkin Elmer Ramanstation equipped with a polarisation head for comparison with spectra obtained at 785 nm (figure S7). The dependence of Raman intensity of the direction of polarisation of the laser was verified by recording Raman spectral along and orthogonal to the optical axis of a calcite Glan Taylor polariser figure S13.

A silicon wafer, placed under the coating as a guide to the depth at which Raman scattering from $[Fe(bipy)_3](BArF)_2$ should be observed (Fig. S9) The intensity of the bands of $[Fe(bipy)_3](BArF)_2$ are much lower and the depth (Z-range) where the signal is observed is deeper then when measured with the PMMA layer on top (*vide supra*). The range over which the $[Fe(bipy)_3](BArF)_2$ was observed increased from roughly 3 μm to 20 μm. The glass slide causes spherical aberration due to a mismatch in refractive index of the glass slide and air, which results in a lower Z-resolution due to the stretching of the confocal volume. Using ITO coated cover slips

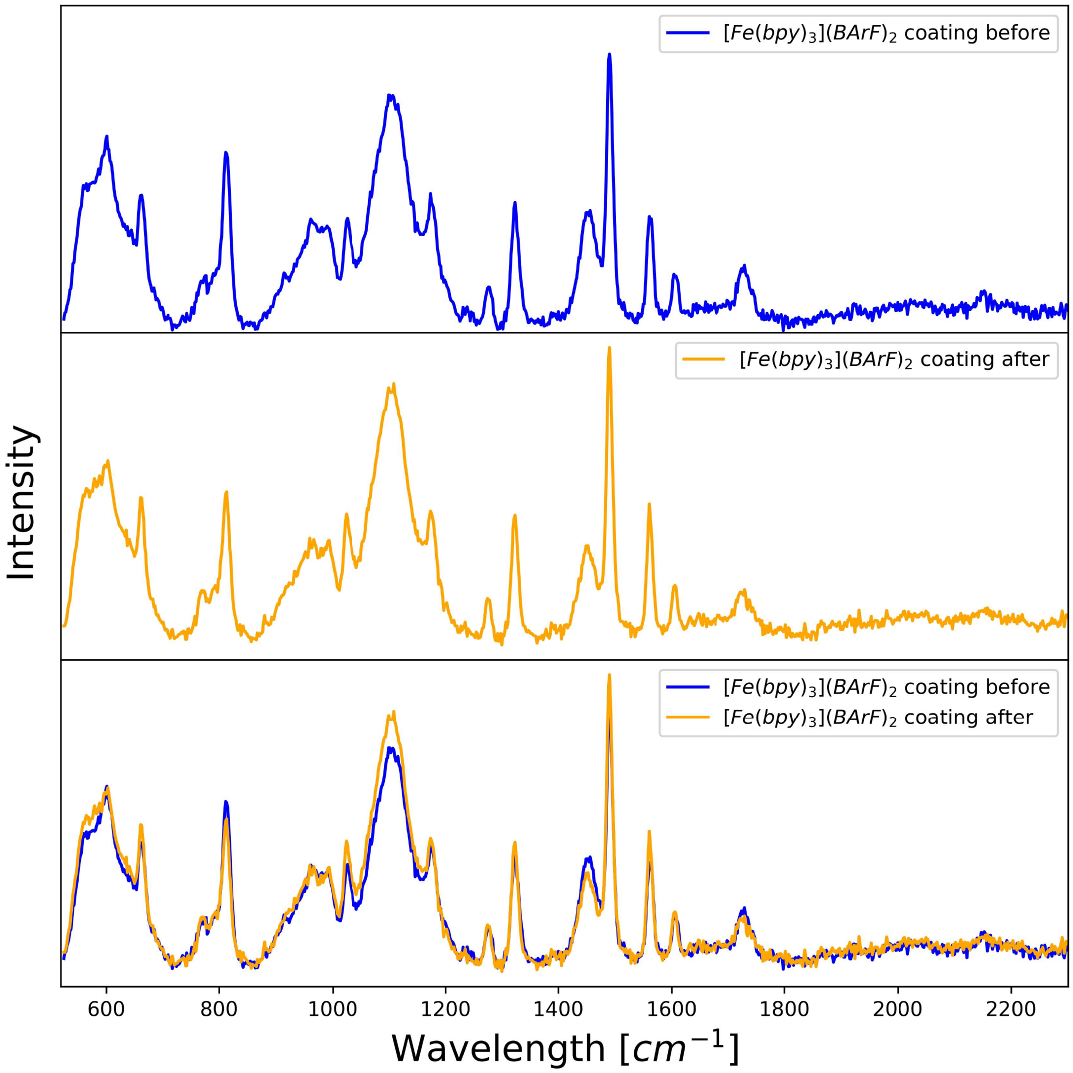

Fig. S2 Raman spectra of a spin-coated PMMA coating with $[Fe(bipy)_3](BArF)_2$ before (blue) and after (orange) soaking in brine for 1 h.

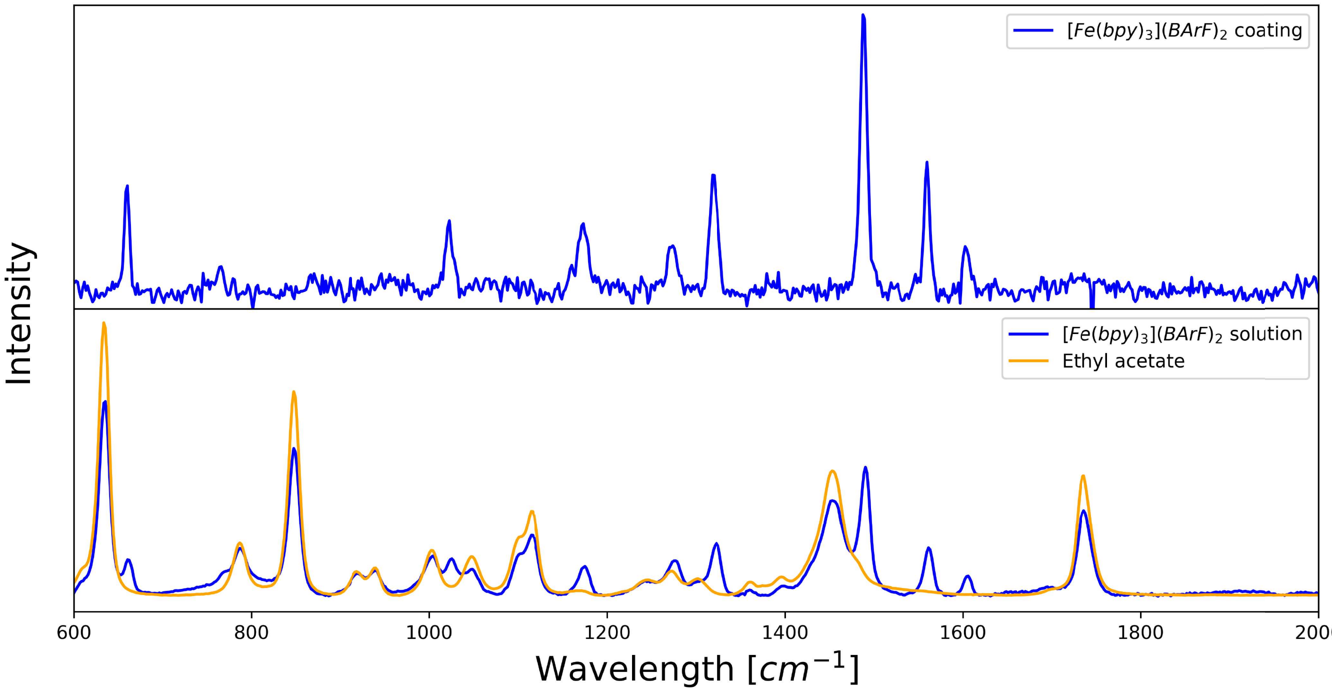

Fig. S3 Raman spectra (at λ_{exc} 532 nm) of (upper) a PMMA film with $[Fe(bipy)_3](BArF)_2$ and (lower) ethyl acetate and a solution of $[Fe(bipy)_3](BArF)_2$ (290 μM) in ethyl acetate

reduced this aberration however, making it possible to distinguish the Raman bands of $[Fe(bipy)_3](BArF)_2$ from the Raman bands of the liquid crystal (*vide infra*).

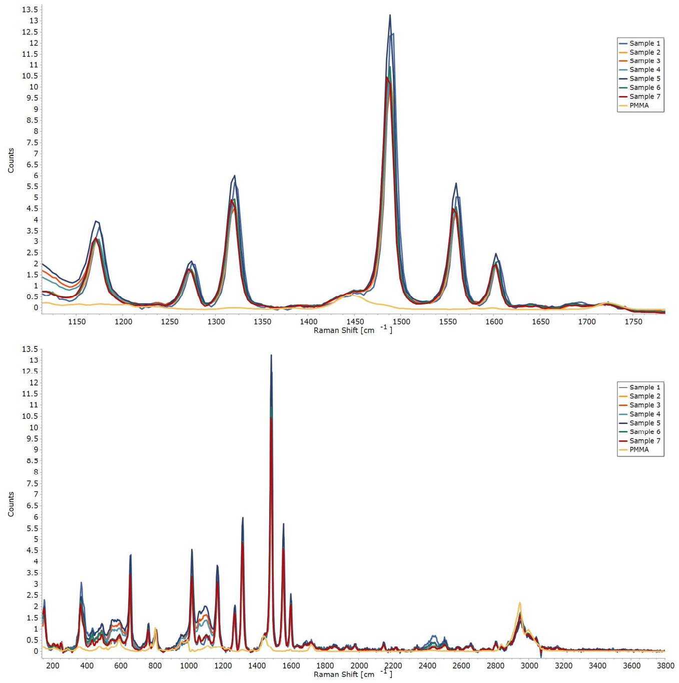

Fig. S4 Spectrum of $[Fe(bipy)_3](BArF)_2]$ in PMMA recorded at 7 distinct points over the film. The spectrum of PMMA is shown in yellow for comparison. Spectra were normalised to the C-H stretching band of PMMA

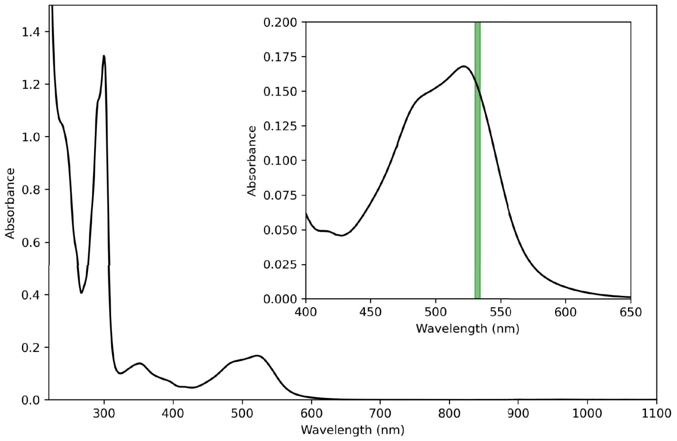

Fig. S5 UV/vis absorption spectra of $[Fe(bipy)_3](BArF)_2$ in acetonitrile (71 μM), 532 nm is indicated with a green line

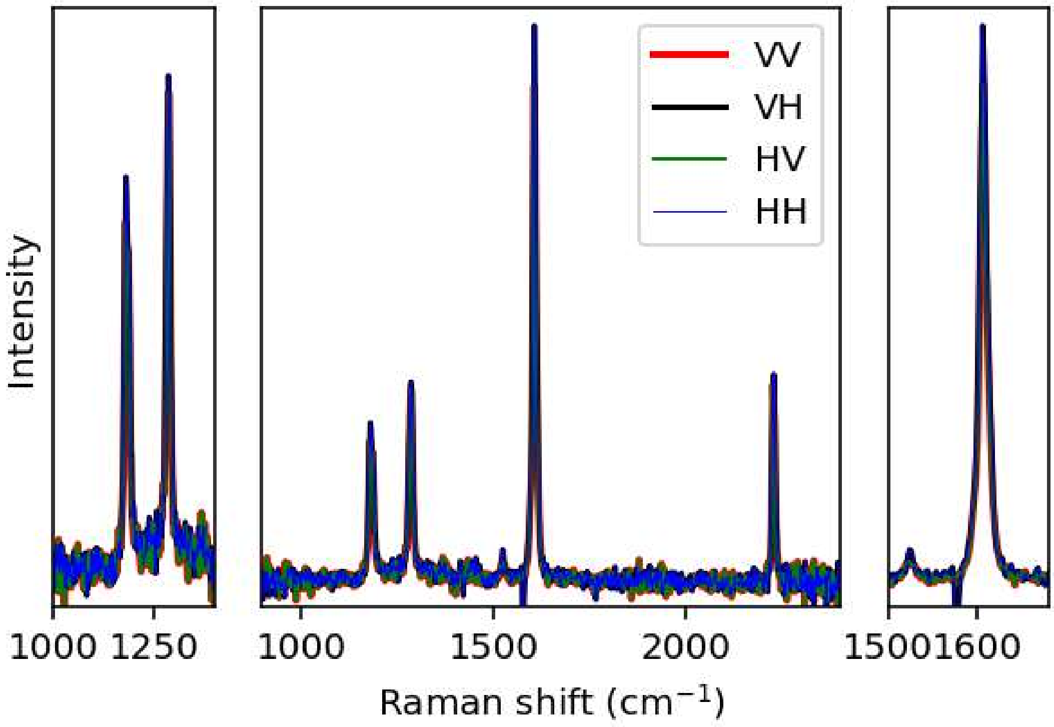

Fig. S6 Raman scattering of 5CB as an isotropic liquid droplet with polarisation laser/collection of v/v, v/h, h/v and h/h.

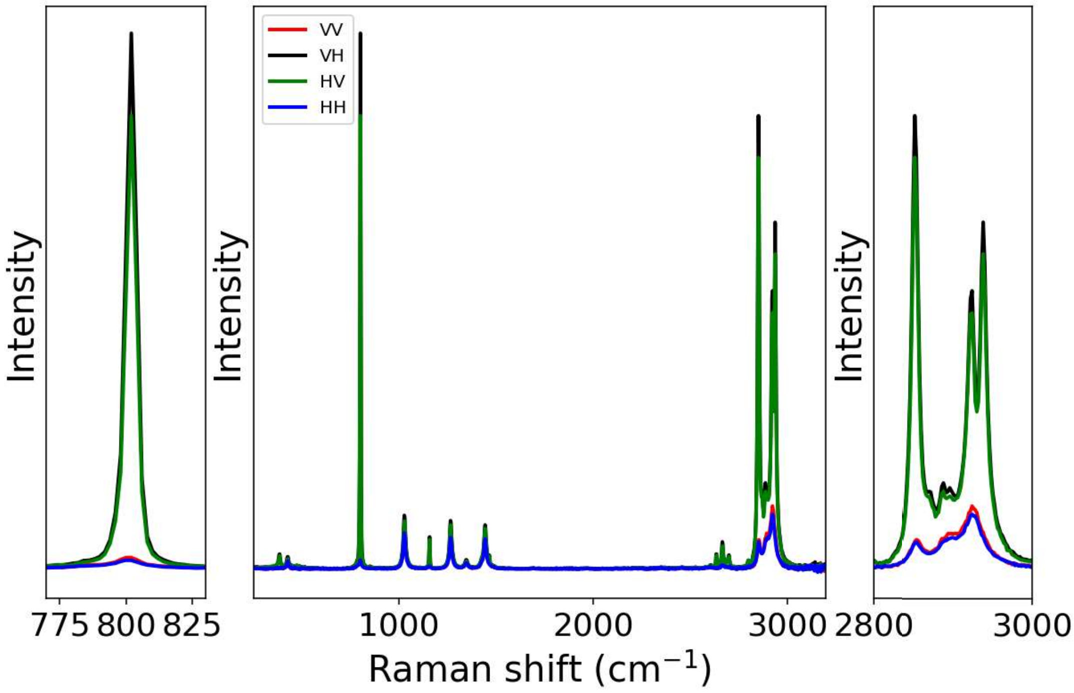

Fig. S7 Polarisation dependence of Raman scattering of cyclohexane.

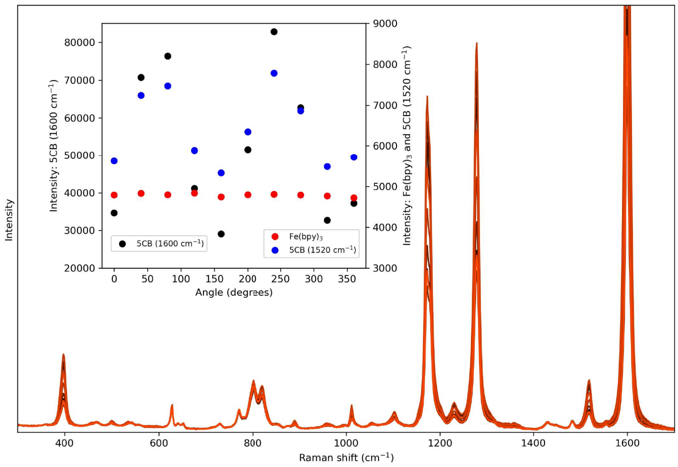

Fig. S8 Confocal Raman spectra of a liquid crystal cell using a 25 μm pinhole. The cell configuration is as in figure 6. Intensity of $[Fe(bipy)_3]^{2+}$ and 5CB (1520 and 1600 cm^{-1}) Raman bands over a range of angles of polarisation of the 532 nm laser. The z-focus was optimised to maximise the intensity of the bands of $[Fe(II)(bipy)_3](BArF)_2$.

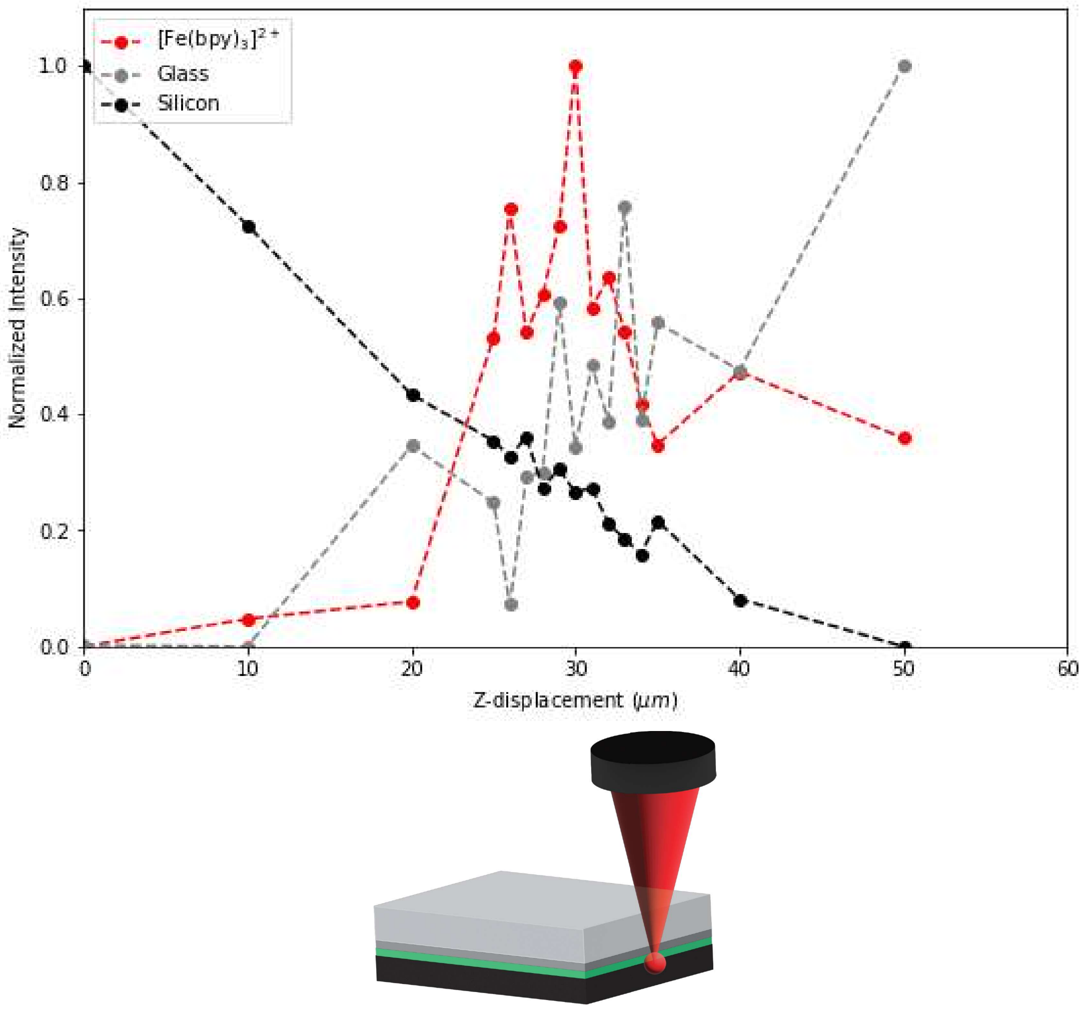

Fig. S9 The sample is arranged such that the glass slide (light grey) coated with [Fe(bipy)$_3$](BArF)$_2$ in PMMA (green) is upside down resting on a silicon wafer (black).

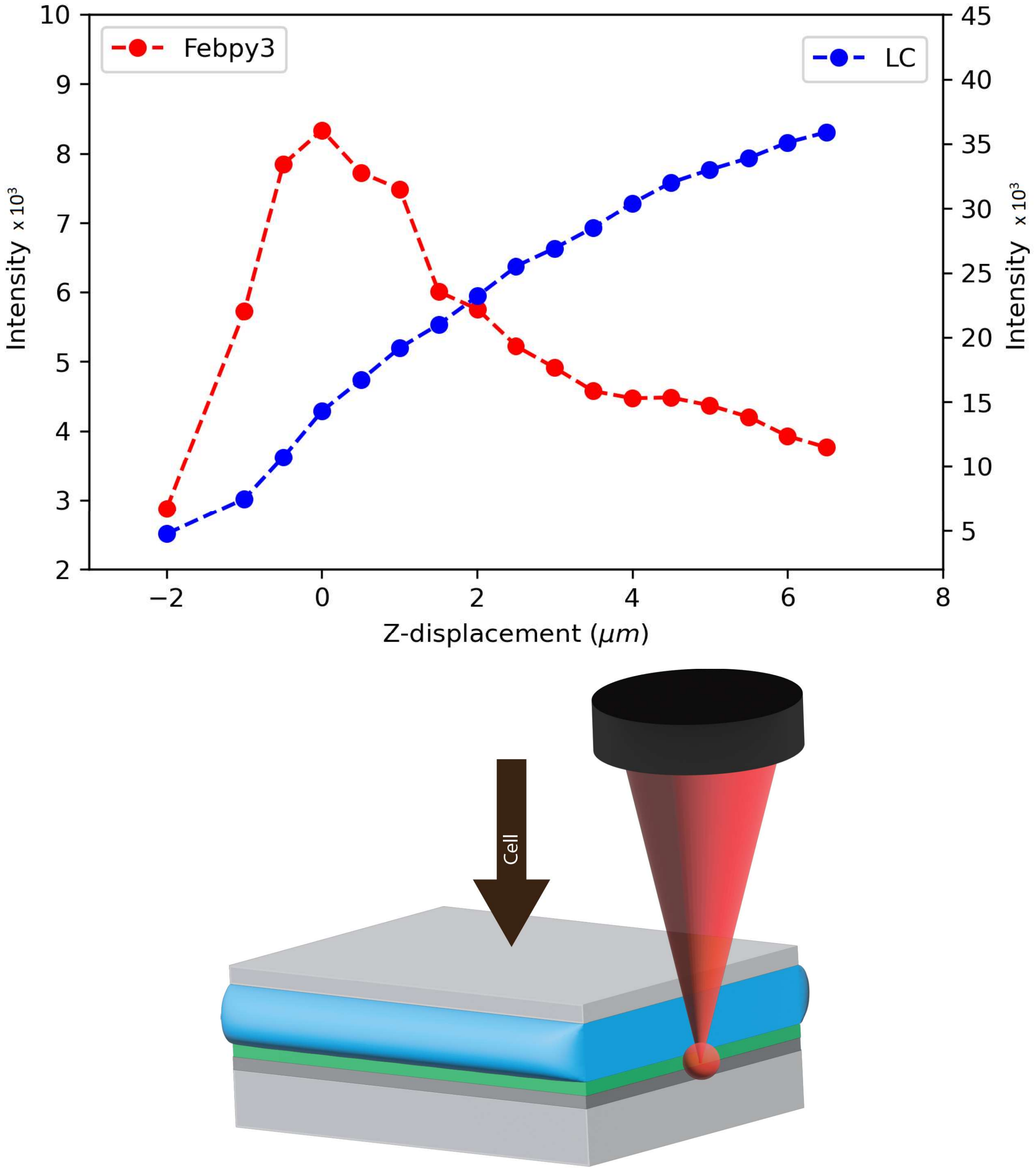

Fig. S10 Z-mapping of a liquid crystal cell using a 25 μm pinhole. The cell configuration is as shown under (and in figure 6) and shows the repeatability of the measurement. The LC cell is assembled with a layer of PMMA/[Fe(bipy)$_3$](BArF)$_2$ (green) spin coated on ITO coated (dark grey) glass (light grey), a 6 μm layer of 5CB (blue) and on top an ITO-coated quartz- cover slip (light grey). The intensity of [Fe(bipy)$_3$]$^{2+}$ and 5CB Raman bands over a range of depths, with cover slip on top. The maximum intensity of the bands of [Fe(II)(bipy)$_3$](BArF)$_2$ is taken as the zero point in the depth scan.

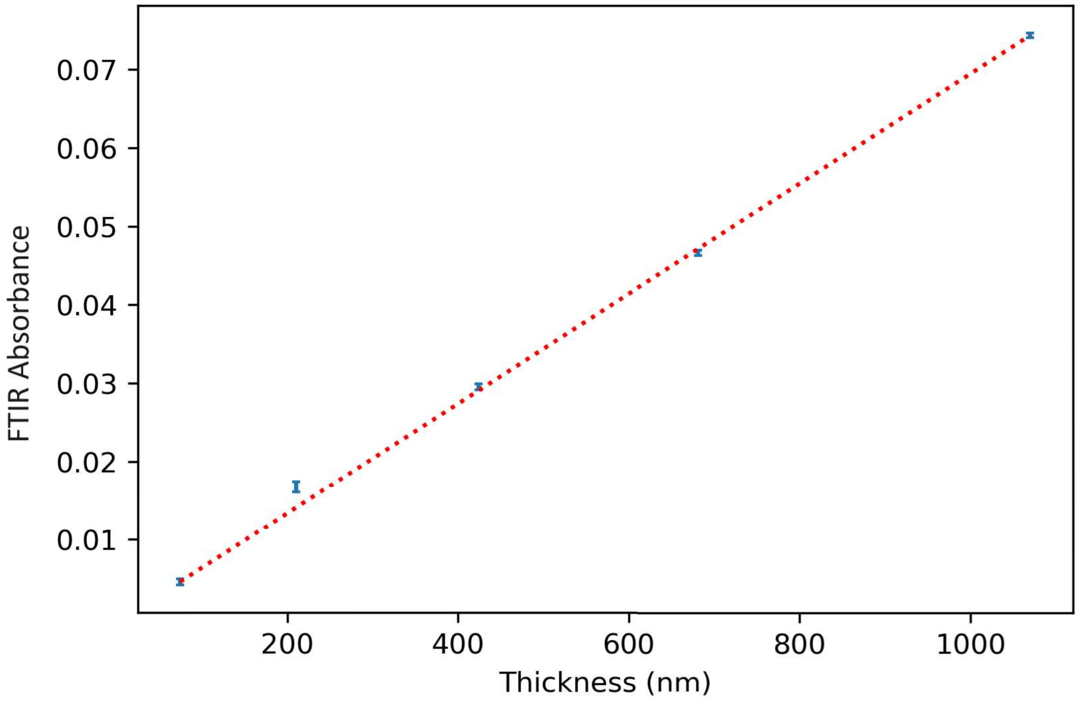

Fig. S11 Calibration curve for determination of thickness of PMMA coatings by FTIR spectroscopy

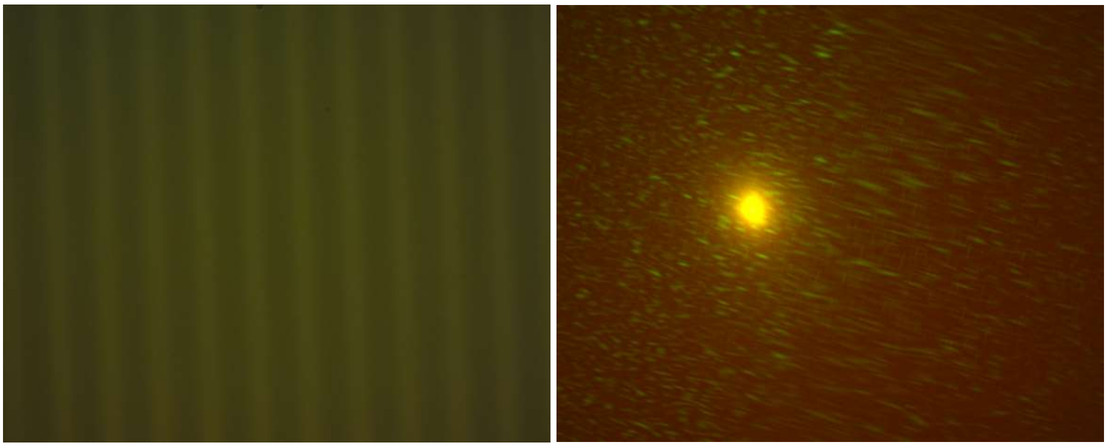

Fig. S12 Ruler without and with illumination with the 532 nm laser to determine maximum spot diameter

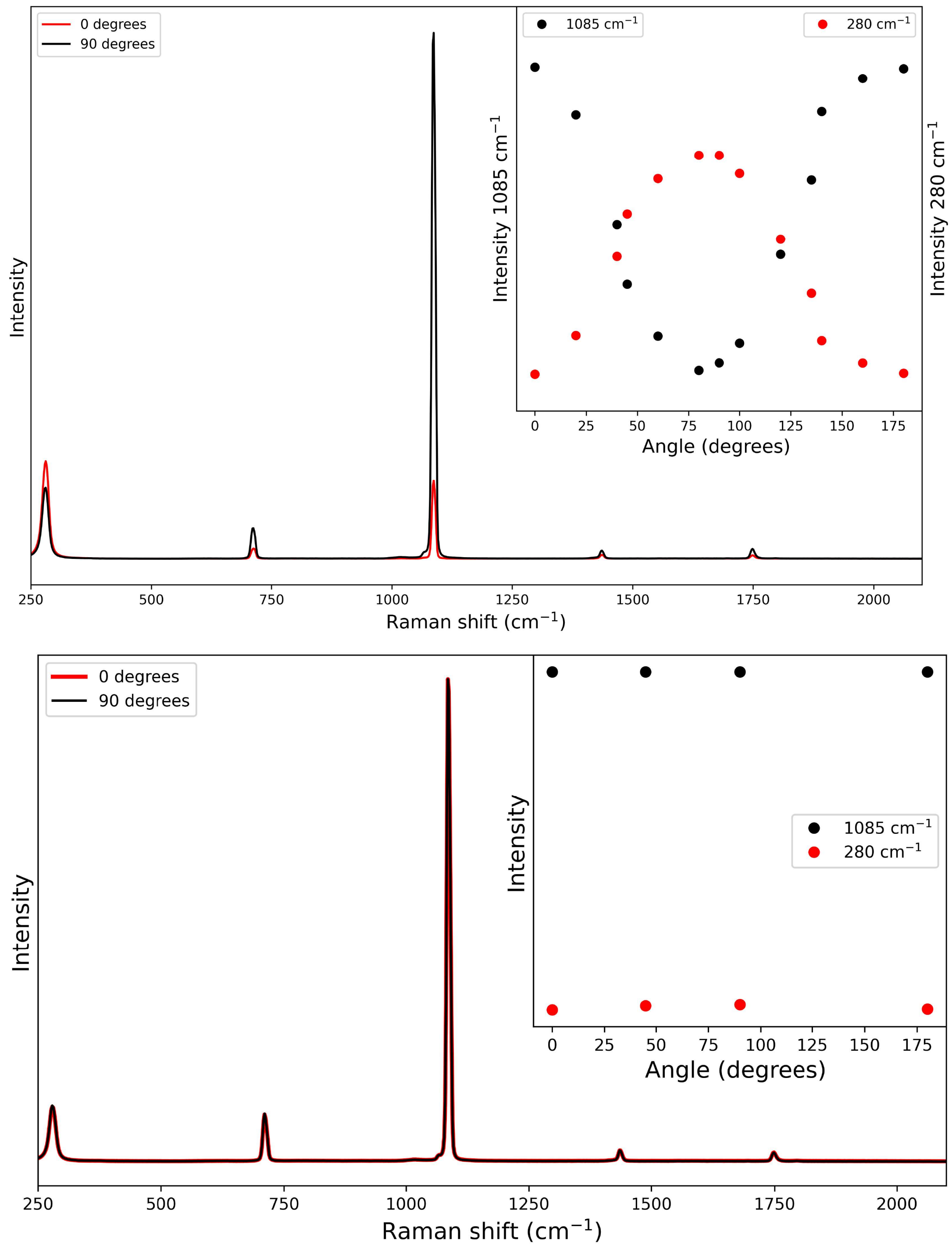

Fig. S13 Dependence of Raman scattering intensity of Glan Taylor (calcite) polariser on the orientation with respect to the polarisation of the laser beam incident (top) orthogonal to (bottom) along the optical axis.

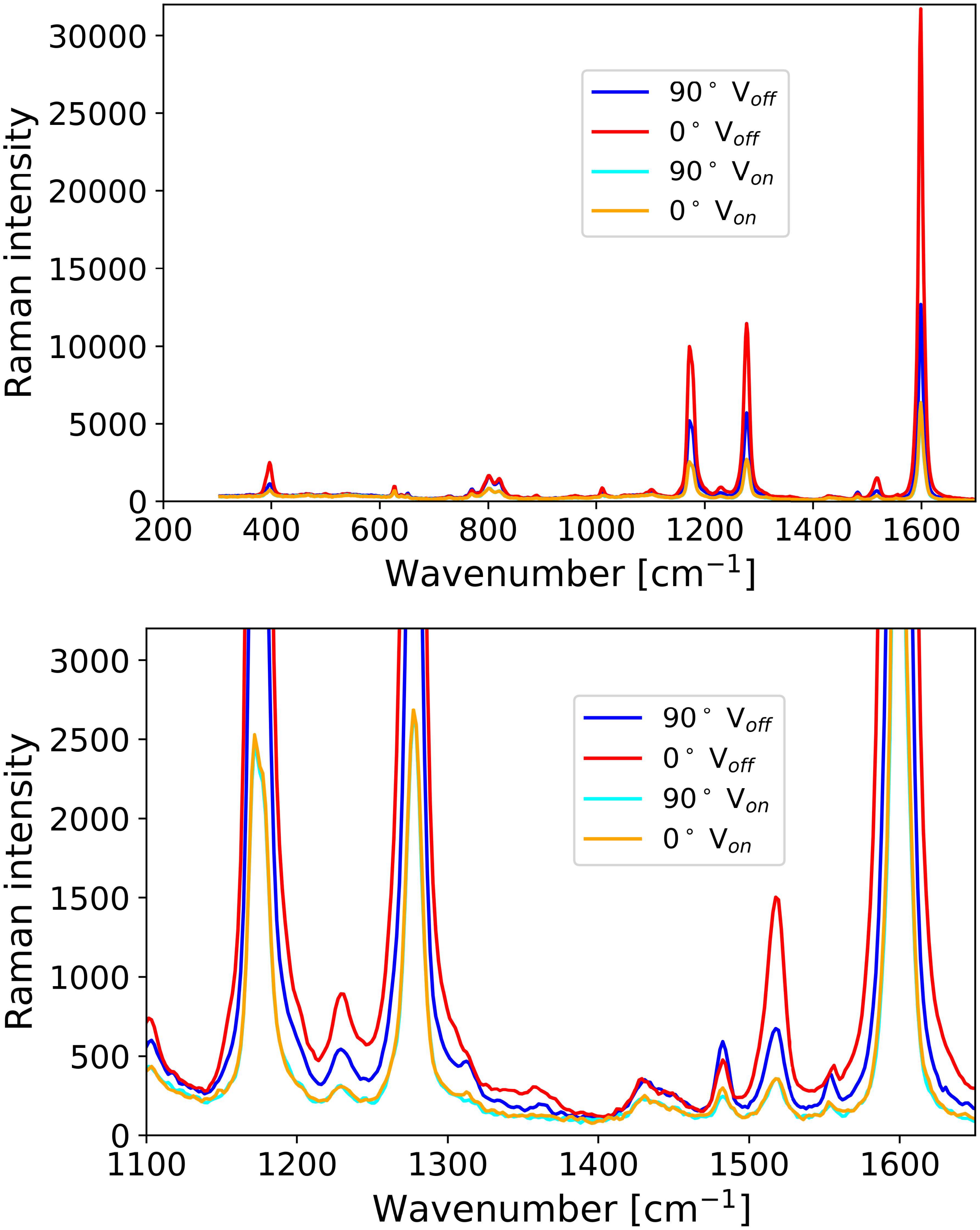

Fig. S14 Confocal Raman spectra of a liquid crystal cell using a 25 μm pinhole with and without application of an alternating voltage across the cell. The cell configuration is as in Fig. 6. Spectra recorded with polarisation of laser along (0°, red and orange) and orthogonal (90°, blue and cyan) to the axis of alignment. Spectra are normalised to the band at 625 cm^{-1}.

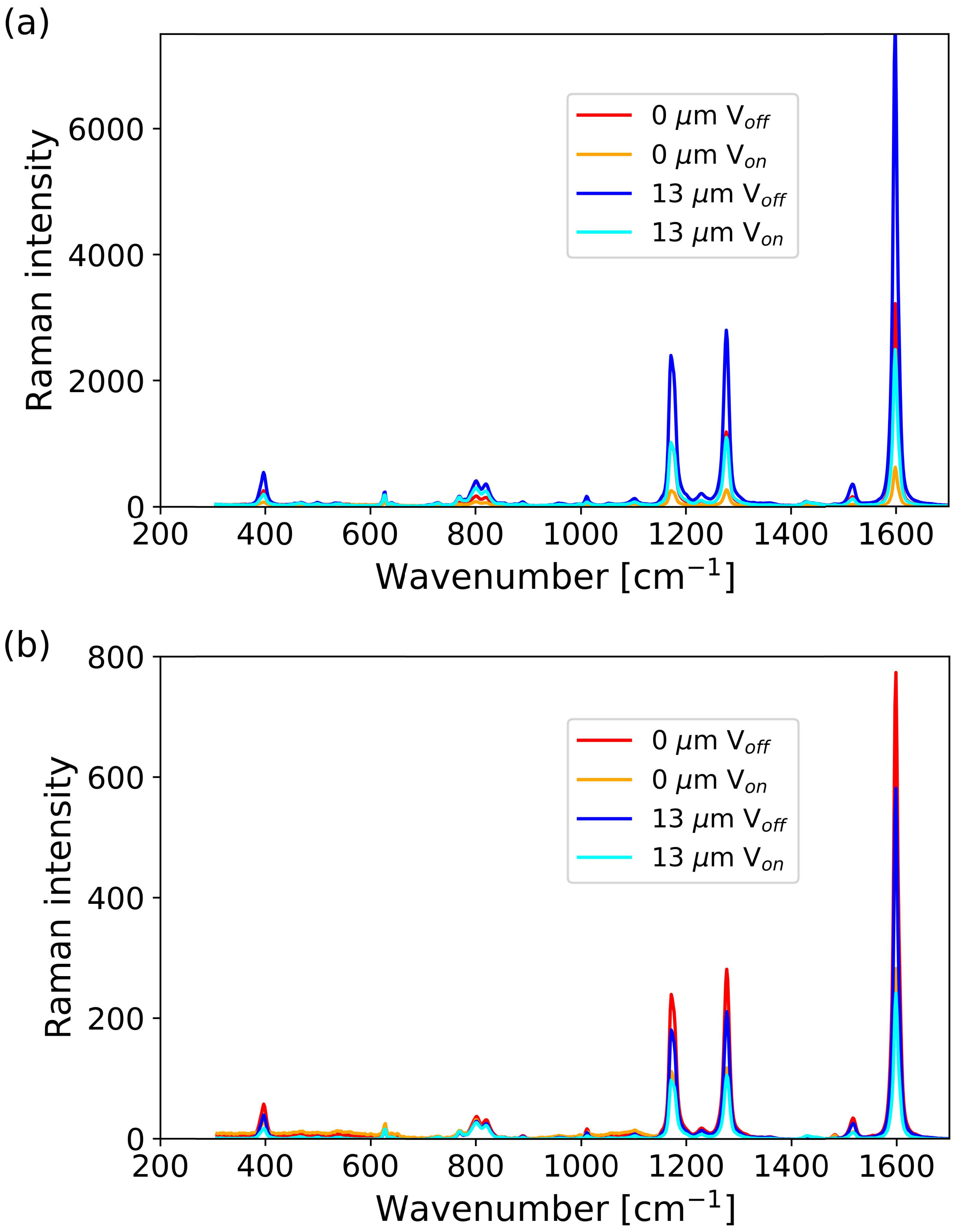

Fig. S15 Confocal Raman spectra of a liquid crystal cell using a 25 μm pinhole. The cell configuration is as in fig. 6. Spectra recorded with polarisation of laser along (0°) the axis of alignment at 0 and 13 μm depths. Spectra before (a) and after (b) normalisation to the band at 625 cm^{-1}.
